# Graph Attention Network and Informer for Multivariate Time Series Anomaly Detection

**DOI:** 10.3390/s24051522

**Published:** 2024-02-26

**Authors:** Mengmeng Zhao, Haipeng Peng, Lixiang Li, Yeqing Ren

**Affiliations:** 1 Information Security Center, State Key Laboratory of Networking and Switching Technology, Beijing University of Posts and Telecommunications, Beijing 100876, China; zhaomeng777@bupt.edu.cn (M.Z.); lixiang@bupt.edu.cn (L.L.); yeqing_ren@bupt.edu.cn (Y.R.); 2 National Engineering Laboratory for Disaster Backup and Recovery, Beijing University of Posts and Telecommunications, Beijing 100876, China; 3 Department of Information Science and Engineering, Zaozhuang University, Zaozhuang 277160, China

**Keywords:** anomaly detection, mutlivariate time series, graph attention network, Informer, industrial control systems

## Abstract

Time series anomaly detection is very important to ensure the security of industrial control systems (ICSs). Many algorithms have performed well in anomaly detection. However, the performance of most of these algorithms decreases sharply with the increase in feature dimension. This paper proposes an anomaly detection scheme based on Graph Attention Network (GAT) and Informer. GAT learns sequential characteristics effectively, and Informer performs excellently in long time series prediction. In addition, long-time forecasting loss and short-time forecasting loss are used to detect multivariate time series anomalies. Short-time forecasting is used to predict the next time value, and long-time forecasting is employed to assist the short-time prediction. We conduct a large number of experiments on industrial control system datasets SWaT and WADI. Compared with most advanced methods, we achieve competitive results, especially on higher-dimensional datasets. Moreover, the proposed method can accurately locate anomalies and realize interpretability.

## 1. Introduction

Industrial control systems are vital to the development of society. Typical ICS such as power plants and sewage treatment plants are being connected to external networks for remote access information, which increases the risk of being attacked. It is important to determine time series anomalies generated by sensors or controllers in time to ensure ICS security.

At present, fruitful research results have been achieved by means of multivariate time series anomaly detection. They can be divided into classical methods and deep learning-based methods. Methods based on classical approaches have been proposed, including wavelet-based [1,2], ARIMA-based [3,4], distance-based [5,6,7], and so on. Certain results regarding modeling time series have been achieved with statistical and mathematical methods. However, with an increase in time series feature dimension, it is difficult to model complex time series features well with these classical methods.

In view of deep learning’s excellent performance in solving various complex problems, it is also widely used in feature learning and time series anomaly detection, for instance, the  methods based on Recurrent Neural Network (RNN) [8], Convolutional Neural Network (CNN) [9,10], Long Short-Term Memory (LSTM) [11,12], and Autoencoder (AE) [13,14,15,16,17,18,19,20,21,22]. In [23,24], time series anomaly detection is realized by using VAE to construct a data distribution. Moreover, some researchers [25,26,27] have used generators and discriminators to model features in time series. Although the methods based on deep learning have improved accuracy compared with the classical methods, it is difficult to obtain the relationships existing in multivariate time series with these methods.

Graph neural networks [28,29,30,31] apply different graph structures to learn the relationships in time series. Deng and Hooi [29] proposed a forecasting-based Graph Deviation Network (GDN) method to detect anomalies by using graph attention. Although the interrelationship can be learned via GDN in different time series and favorable prediction effect can be achieved, GDN does not perform well on higher-dimensional data. Moreover, the anomaly detection methods based on forecasting only focus on short time series forecasting and do not consider the impact of future trends.

To solve the above-mentioned problem, we employ graph attention and Informer for anomaly detection. Graph Attention Network is used to learn interrelationships of time series, and Informer is used to learn features of long time series. For short-time series forecasting, we add Gated Recurrent Unit (GRU) to GAT to better learn the characteristics of the time series. In addition, we apply long time series forecasting to assist short time series to obtain the forecast value of the next moment, and finally determine whether the time series is abnormal or not. Overall, the main contributions of our work are presented as follows:

- A fresh method based on graph attention and Informer is proposed for multivariate time series anomaly detection; the method is based on time series forecasting.
- We employ graph attention and GRU to learn the short-term features of time series and Informer to learn the long-term features of time series. Then, we use long time series forecasting to guide the short time series prediction and complete the final anomaly judgment.
- To improve the accuracy of the model, the loss of short-term forecasting and the loss of long-term forecasting are considered.
- Experiments on SWaT and WADI datasets show that our model has higher time series anomaly detection performance.

The rest of this article is organized as follows. Section 2 reviews time series anomaly detection methods. Section 3 details the proposed scheme, graph attention-based short time series forecasting, Informer-based long time series forecasting, model optimization, and anomaly scoring. Section 4 is the experimental evaluation of the model. The conclusion and the future work are in Section 5.

## 2. Related Work

Many anomaly detection methods have been proposed, as shown in Table 1; we will discuss anomaly detection schemes based on classical methods and deep learning-based methods.

### 2.1. Classical Methods

Classical methods using statistical and mathematical methods model the time series distribution. In [32], principal component analysis was applied to anomaly detection, and the classifier consists of major principal components and minor principal components for normal instances. Liu et al. [33] first proposed iForest according to the characteristics that anomalies are different and few; anomalies are recognized by shorter paths in comparison with normal instances. Lu and Ghorbani [1] applied wavelet coefficients as the input of the model and made decisions by outlier detection algorithm according to the difference. Yaacob et al. [3] proposed ARIMA based on the previous data to predict the expected normal traffic, and anomaly detection was realized by comparing the predicted traffic with the actual traffic. Boniol et al. [7] proposed SAND, which constructs subsequence datasets and weighting by using statistical features, and their model based on the distance to normal behavior to detect anomalies. Although a variety of classical methods have been proposed, there are shortcomings, including low detection rate and inability to obtain the correlations between multivariate time series.

### 2.2. Deep Learning-Based Methods

Deep learning-based time series anomaly detection methods can be categorized into reconstruction-based and forecasting-based methods.

#### 2.2.1. Reconstruction-Based Methods

With the methods on reconstruction, the characteristics of time series through model training can be learned, and whether there is an anomaly according to the reconstruction error can be judged. OmniAnomaly [8] utilized random variables to obtain normal patterns, and then reconstruction probabilities are used to identify anomalies. For AE anomaly detection, Encoder was employed to compress the features and restored the time series through the Decoder [14,18,19,20]. An anomaly detection algorithm method based on VAE was proposed in [23,24,34]. In [25,26,27,35], different Generative Adversarial Networks enable us to obtain the features of multivariate data, and the results reconstructed via the generator are applied to realize the time series anomaly detection. Recently, with some methods, Transformer has been employed to learn the features of time series, and anomaly detection has been realized through reconstruction [36,37,38,39,40]. Although certain anomaly detection effects have been achieved with the method based on reconstruction, we pay more attention to the time series methods based on forecasting as they are more intuitive and easier to understand.

#### 2.2.2. Forecasting-Based Methods

With the methods based on forecasting, the value of the next moment through historical data can be predicted, and whether there is an anomaly according to the predicted value and the observed value can be determined. The anomaly detection method DeepAnT [9] utilized deep CNN for forecasting and realized anomaly judgment through the anomaly detector. In [11], LSTM was employed for time series anomaly detection. Zhang et al. [18] proposed an anomaly detection method via Deep Convolutional Autoencoding Memory network, and the method leverages a Deep Convolutional Autoencoder, a memory network to obtain the features of time data, and achieves the purpose of forecasting.

Deep learning-based methods effectively improve the performance of time series anomaly detection but fail to obtain the interdependencies. Many time series anomaly detection models based on graph attention have been proposed and achieved good detection results. Zhao et al. [41] proposed graph attention to learn complex dependencies regarding time and feature dimensions of time series, while anomaly detection is achieved using prediction and reconstructed models. Graph Deviation Network (GDN) [29] utilizes embeddings to construct graph structures. Graph attention is employed to learn the features of time series, and anomaly scores are obtained based on observed and predicted values, allowing for the judgment of time series anomalies. Building upon the architecture of GDN, GRN [31] significantly enhances the performance of time series anomaly detection by incorporating GRU technology. Moreover, MST-GAT [42] addresses the complexities of multimodal scenarios in multivariate time series by applying graph attention mechanisms both within and between modalities, thereby deeply exploring the features of time series. Given the outstanding performance of GAT in the realm of time series anomaly detection, our model also adopts the graph attention mechanism to precisely learn the features of time series.

The graph-based time series anomaly detection method of GDN [29] uses graph structure to represent the interrelationships between time series and has a high detection rate, but its performance in F1 and recall indicators is poor. At the same time, as for this method, the influence of future time series trends on time series has not been taken into consideration. We use GRU in our model to better learn time series characteristics due to its favorable performance in learning time series features. In [41], an anomaly detection method based on forecasting and reconstruction was proposed; the characteristics of short and long time series have been taken into consideration. Miao et al. [43] proposed a time series anomaly detection method based on short-term and long-term mask representation learning. However, Informer [44] is a variant of Transformer that has favorable performance in long time series forecasting. This inspired us to use both graph attention and Informer for time series anomaly detection.

**Table 1:** Summary of related work.

Classification	References	Description	Disadvantage
Classical methods	[1,3,7,32,33]	Using statistical and mathematical methods, model the time series distribution	Low detection rate and inability to obtain the correlations between multivariate time series
Deep learning methods (excluding graph neural networks)	[8,9,10,11,12,13,14,15,16,17,18,19,20,23,24,25,26,27,34,35]	Deep learning-based time series anomaly detection methods can be categorized into reconstruction-based and forecasting-based methods	Unable to capture the internal relationships within the time series
Graph neural networks methods	[29,31,36,44]	Graph network is used to learn complex dependencies on time and feature dimensions of time series	The performance decreases sharply with the increase in feature dimension, and the influence of future time series trends on time series has not been taken into consideration

## 3. Methodology

### 3.1. Problem Formalization

In this work, $\mathrm{MS}=[x^{1},x^{2},\cdot \cdot \cdot ,x^{T}]$ is the time series generated by data source *M* over *T* periods, where $\mathrm{MS}\in R^{M\times T}$, $x^{t}\in R^{M}$. Our goal is to design a model displaying the features and regularities of data so that the model can detect anomalies from time series.

At time *i*, we utilize the sliding window *l* to obtain input data $S_{i}=\left[x^{i-l},\cdot \cdot \cdot ,x^{i-2},x^{i-1}\right]\;$ from X, i≥l, $S_{i}\in R^{M\times l}$. With this model, we obtain the predicted value ${\hat{S}}_{i}=\left[{\hat{x}}^{i}\right]$ according to historical data $S_{i}$ and determine whether the observed value $x^{i}$ is normal (0) or abnormal (1) according to the forecasted ${\hat{x}}^{i}$.

### 3.2. Overall Scheme

**Figure 1:**
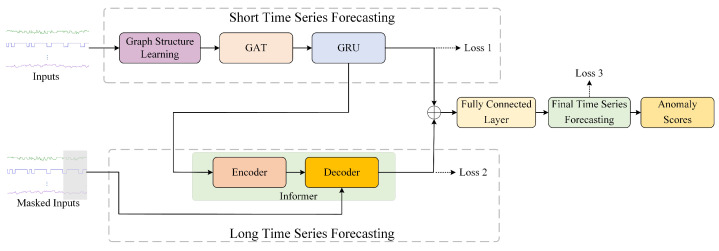
Overview of the proposed framework. The time series are fed into the time series forecasting modules. The model calculates the anomaly score based on the prediction results.

We propose time series feature learning based on graph neural network and Informer, which realizes long-time forecasting and short-time forecasting. As shown in Figure 1, our proposed framework mainly includes the following four parts:

(1) Graph attention-based short time series forecasting. Graph Attention Network and GRU are employed to learn characteristics of time series, and then we predict the next values of the time series.
(2) Long time series forecasting based on Informer. Informer is used to implement time series forecasting for the next period.
(3) Joint optimization model. Short time series forecasting and long time series forecasting are jointly optimized to elicit the final time series forecasting.
(4) Anomaly scoring. We calculate the anomaly score of the time series, and appropriate threshold is selected to obtain the judgment of whether the time series is abnormal.

### 3.3. Graph Attention-Based Short Time Series Forecasting

Although each data source generates the time series in parallel, there is a certain relationship among these time series. For instance, in terms of sewage treatment plant, the level of a tank determines the inflow and outflow. We employ a directed graph structure to represent the interrelationships among time series, apply graph attention to learn the characteristics of time series, and then achieve short-term prediction of time series.

#### 3.3.1. Construction of Graph Structure

Before using graph attention for time series feature learning, we build a graph structure *W* through the embeddings of time series. The embedding vector for the *i*-th data source is denoted as $u_{i}\in R^{d},i\in \{1,2,\cdot \cdot \cdot ,M\}$, and *M* is the total number of data sources (i.e., features), with each data source represented by a *d*-dimensional vector. These embedding vectors are initially generated randomly and are continuously optimized and updated during model training to more accurately capture the characteristics of the time series. The graph structure *W* is generated using the following formula.

(1) $$w_{ji}=\frac{{u_{i}}^{⊤}u_{j}}{\|u_{i}\left\|\cdot \right\|u_{j}\|}\;\mathrm{for}\;j\neq i,$$

(2) $$W_{ji}=1\left\{j\in \mathrm{TopK}\right\}.$$

By calculating the normalized dot product $w_{ji}$ between embedding vector *i* and other embedding vectors *j*, we can measure the correlation between two data sources. The adjacency matrix *W* is used to describe the connections between nodes in the graph structure, with $W_{ji}$ = 1 indicating a directed edge from node *j* to *i* and $W_{ji}$ = 0 indicating no connection between the two nodes. Given that only a subset of data sources needs to communicate with each other, we select the TopK largest values in $w_{ji}$ and set the corresponding $w_{ji}$ to 1, thus constructing a sparse directed graph.

#### 3.3.2. Information Aggregation Based on Graph Attention

After obtaining the graph structure, we apply graph attention to realize the aggregation of information. Different from the existing graph attention mechanism, the features embedded by the corresponding data source are added during feature learning. All nodes pointing to *i* for information aggregation are represented by $P_{i}$ as follows:

(3) $$P_{i}^{t}=ReLU\left(\alpha_{i,i}Hs_{i}^{t}+\sum_{j\in U(i)}\;\alpha_{i,j}Hs_{j}^{t}\right),$$

where $s_{i}^{t}\in R^{l}$ is the input feature for node *i*, the trainable weight matrix $H\in R^{d\times l}$, $U\left(i\right)=\left\{j|W_{ji}>0\right\}$ is the nodes points to *i* in the neighbor matrix *W*, and $\alpha_{i,j}$ is calculated by the following formula:

(4) $$g_{i}^{t}=u_{i}⊕Hs_{i}^{t},$$

(5) $$\pi \left(i,j\right)=LeakyReLU\left(a^{⊤}\left(g_{i}^{t}⊕g_{j}^{t}\right)\right),$$

(6) $$\alpha_{i,j}=\frac{\exp(\pi (i,j))}{\sum_{k\in U\left(j\right)\cup \left\{i\right\}}\exp\left(\pi \left(i,k\right)\right)},$$

where ⊕ denotes concatenation, $g_{i}^{t}$ is the connection of the data source embeddings $u_{i}$ and the corresponding transformed features $Hs_{i}^{t}$, *a* is learned attention mechanism coefficients. We apply Softmax to normalize the attention coefficients, and when calculating attention coefficients we utilize LeakyReLU as nonlinear activation.

#### 3.3.3. Short Time Series Forecasting

After the time series feature learning through Graph Attention Network, we employ GRU to learn the characteristics of the time series. We multiply the output $z_{i}$ of the GRU with the corresponding embedding $u_{i}$ (denoted as ×), and then all results are stacked together and inputted to the fully connected layer $f_{\theta }$ to obtain the predicted value of the next moment, which is shown as follows:

(7) $${\hat{S}}_{K}=f_{\theta }\left(\left[u_{1}\times z_{1}^{\left(t\right)},\cdot \cdot \cdot ,u_{M}\times z_{M}^{\left(t\right)}\right]\right),$$

We employ the Mean Squared Error (MSE) loss function to minimize the predicted output ${\hat{S}}_{K}$ and the observed value $S_{K}$:

(8) $$Loss_{s}=\|{\hat{S}}_{K}-S_{K}{\|}^{2}.$$

### 3.4. Long Time Series Forecasting Based on Informer

**Figure 2:**
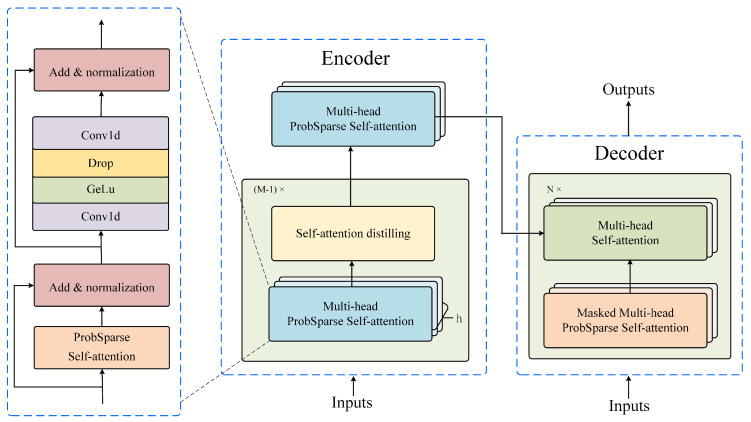
The process of Encoder and Decoder. The model is based on a Transformer with ProbSparse self-attention. The output from the previous module (the short-term time series prediction module) is input into the Encoder, while the masked input is fed into the Decoder, achieving the encoding and decoding of the sequence.

We apply Informer in long time series forecasting, which includes Encoder and Decoder. We show the process of Encoder in Figure 2, where the main components are ProbSparse self-attention and self-attention distilling proposed by [44]. We first describe the process of long time series prediction and then introduce the two important components.

#### 3.4.1. Long Time Series Forecasting

The specific process of long-term series forecasting is shown in Figure 2. For Encoder, the input $X^{t}\in R^{l\times M}$ is the output of GRU, and then $X^{t}$ is embedded by 1D convolution and position encoding, respectively. Both outputs are added together to obtain $X_{\mathrm{en}}^{t}\in R^{l\times d_{\mathrm{model}}}$. $X_{\mathrm{en}}^{t}$ is input into the network structure composed of (N−1) layer ProbSparse self-attention and self-attention distillation, and then output result is input into the ProbSparse self-attention.

For Decoder, the input $X_{\mathrm{de}}^{t}={\mathrm{Concat}(X}_{\mathrm{token}}^{t}{,X}_{0}^{t})\in R^{\left(L_{token}+L_{y}\right)}$ is a combination of $X_{\mathrm{token}}^{t}$ and $X_{0}^{t}$, where $X_{\mathrm{token}}^{t}\in R^{L_{token}}$ is the historical data, $X_{0}^{t}\in R^{L_{y}}$ is the time series replaced by 0, $L_{token}$ is the length of the input to Decoder, $L_{token}$ is less than *l*, $L_{y}$ is the length of long-term series forecasting. Like Encoder, the transformation of input is realized through Conv1D mapping and positional encoding position embedding, respectively. Decoder is a network structure composed of *N*-layer probability sparse self-attention and traditional self-attention layer. Different from the sparse self-attention in Encoder, sparse self-attention with mask is employed, which prevents learning information behind the current position by making the dot product negative infinity. Finally, by using fully connected layer, we obtain the predicted time series ${\hat{S}}_{j}=\left[{\hat{S}}_{j}^{1},{\hat{S}}_{j}^{2},\cdot \cdot \cdot ,{\hat{S}}_{j}^{L_{y}}\right]$. The optimization of long time series forecasting can be realized by the MSE loss function as follows:

(9) $$Loss_{L}=\|{\hat{S}}_{j}-S_{j}{\|}^{2}.$$

#### 3.4.2. ProbSparse Self-Attention

ProbSparse self-attention is an improved self-attention method. The main formula is provided as follows:

(10) $$\mathrm{Att}(Q,K,V)=\mathrm{Softmax}(\frac{\bar{Q}K^{⊤}}{\sqrt{d}})V,$$

where *Q*, *K*, *V* are the input vector X learned by different matrices *W*, $Q\in R^{L_{Q}\times d}$, $K\in R^{L_{K}\times d}$, $V\in R^{L_{V}\times d}$. *d* is the input dimension, $\bar{Q}$ is a sparse matrix of the same size as *q*, which is obtained by the largest *q* queries sparsity measure $M(q_{i},K)$.

The sparsity metric $M(q_{i},K)$ is formulated as follows:

(11) $$M\left(q_{i},K\right)=\max_{j}\;\left\{\frac{q_{i}k_{j}^{⊤}}{\sqrt{d}}\right\}-\frac{1}{L_{K}}\sum_{j=1}^{L_{K}}\frac{q_{i}k_{j}^{⊤}}{\sqrt{d}},$$

where $k_{j}$ is randomly selected from *K*, and the number is $U=L_{Q}\ln L_{K}$. Then, we calculate the score of $k_{j}$ and $\bar{Q}$ and select top-*h* sequences from $M(q_{i},K)$ to form *Q*, $h=c\cdot \ln L_{Q}$, *c* is the hyperparameter. The *Q* of each head calculates the attention except for the top-*h* sequences, and the remaining *Q* corresponding attention is replaced by the average of the *V*, and then the results of the multi-head attention are merged. Different from Feed-Forward of ordinary Transformer, we first apply convolution with GELU activation function to map the data. In our methods, residuals and normalization are used in self-attention and Feed-Forward for better feature extraction.

#### 3.4.3. Self-Attention Distilling

The self-attention distillation layer is shown as follows:

(12) $$X_{j+1}^{t}=\mathrm{MaxPool}(\mathrm{ELU}(\mathrm{Conv}1d(\;\;\;{\left[\;\;\;X_{j}^{t}\right]}_{\mathrm{AB}}))),$$

where ${\;\;\;[\;\;\;\cdot \;\;\;]\;\;\;}_{\mathrm{AB}}$ represents ProbSparse self-attention. Conv1D performs convolution filtering with ELU activation function in the time dimension. MaxPool applies max-pooling layer; the stride is 2, which reduces $X^{t}$ by half after one layer and extracts the main features.

### 3.5. Joint Optimization Model

In our anomaly detection model, different from the existing methods that only focus on next moment time series prediction, we consider not only short-term but also the future trend to guide the time series forecasting. Therefore, the final forecasting is a comprehensive consideration of the next moment and the forecasting for next period. We concatenate the output ${\hat{S}}_{k}=S_{k}^{1}$ and ${\hat{S}}_{j}=\left[{\hat{S}}_{j}^{1},{\hat{S}}_{j}^{2},\cdot \cdot \cdot ,{\hat{S}}_{j}^{L_{y}}\right]$, and then we obtain the final short time series forecasting result ${\hat{S}}_{i}$ by fully connected layer $f_{\theta }$ as follows:

(13) $${\hat{S}}_{i}=f_{\theta }\left(Concat\left({\hat{S}}_{k},{\hat{S}}_{j}\right)\right).$$

The loss function of joint optimization is shown as follows:

(14) $$Loss_{z}=\|{\hat{S}}_{i}-S_{i}{\|}^{2}.$$

The total loss functions are as follows:

(15) $$Loss=\lambda Loss_{s}+\kappa Loss_{L}+\tau Loss_{z},$$

where λ, κ, and τ are hyperparameters. In our experiments, we set λ=κ=τ=1. The training process is summarized in Algorithm 1.
**Algorithm 1:** The training stage of the proposed method**Require:** Original dataset MS; Number epochs *N*Initialized embedding vector *u*, Gat Gat(·),Gru Gru(·), short time series forecasting SF(·), Encoder E(·), Decoder D(·), and fully connected layer $f_{\theta }\left(\cdot \right)$W←un←1**Repeat**   **for** each $S_{i}\in \mathrm{MS}$ **do**:      $X\leftarrow Gru(Gat\left(S_{i},W\right))$      ${\hat{S}}_{K}\leftarrow SF\left(X\right)$      $Loss_{S}=\|{\hat{S}}_{k}-S_{k}{\|}^{2}$      $X_{\mathrm{token}}\leftarrow E\left(X\right)$      $X_{\mathrm{de}}={\mathrm{Concat}(X}_{\mathrm{token}}{,X}_{0})$      ${\hat{S}}_{j}\leftarrow D\left(X_{\mathrm{de}}\right)$      $Loss_{L}=\|{\hat{S}}_{j}-S_{j}{\|}^{2}$      ${\hat{S}}_{i}=f_{\theta }\left(Concat\left({\hat{S}}_{k},{\hat{S}}_{j}\right)\right)$      $Loss_{z}=\|{\hat{S}}_{i}-S_{i}{\|}^{2}$      $Loss=\lambda Loss_{s}+\kappa Loss_{L}+\tau Loss_{z}$      u,Gat(·),Gru(·),E(·),D(·)←update weights using Loss   **end for**n←n+1**until** n=N

### 3.6. Anomaly Scoring

To detect and interpret anomalous time series, we calculate individual scores for each sensor using the trained model, and then at time *t* the following error is computed:

(16) $${\mathrm{Err}}_{i}\left(t\right)=\left|s_{i}^{\left(t\right)}-{\hat{s}}_{i}^{\left(t\right)}\right|,$$

where $s_{i}^{\left(t\right)}$ is the observation value from the i-th sensor at time t, and ${\hat{s}}_{i}^{\left(t\right)}$ is the predicted value from the *i*-th sensor at time *t*.

Because different data sources have different scales, we normalize the anomaly score. The formula is provided as follows:

(17) $$a_{i}\left(t\right)=\frac{{\mathrm{Err}}_{i}\left(t\right)-{\tilde{\mu }}_{i}}{1+{\tilde{\sigma }}_{i}}.$$

We choose the maximum value at each moment as anomaly score. Then, we employ grid search to find out the best F1 score. If the anomaly score is greater than the threshold, we consider the moment to be abnormal. Otherwise, the time series will be regarded as normal.

## 4. Experiments

### 4.1. Datasets

We apply SWaT and WADI in validation of our model. These two datasets are commonly used for time series anomaly detection and are multivariate time series generated from multiple data sources. Both datasets realistically simulate attack scenarios in water treatment plants. The SWaT dataset comes from a water treatment testbed in Singapore [45]. This dataset includes sensor values (water level, flow, etc.) as well as the operation of actuators (valves and pumps). It simulates a modern cyber–physical system and records 51 sensors and actuators operating data for a total of 11 days; normal data were obtained during the first 7 days, and attack data were generated in the last 4 days. WADI is an extension of SWaT, and the dataset contains 14 days of normal data. Over the next 2 days, the system performed some controlled physical attacks at various intervals. Therefore, these data were used in the test set. What needs to be emphasized is that, compared with the 51 features of the SWaT dataset, WADI has 127 features, which is more complex. Table 2 shows the statistics of the two datasets. To speed up the training, we sample every 10 s on SWaT and WADI. The label that appears most frequently within 10 s is used as the label of the data.

### 4.2. Experimental Setup

(1) Evaluation indicators

We apply precision (Pre), recall (Rec), and F1 score to evaluate the performance of our method:

(18) $$\mathrm{Pre}=\frac{\mathrm{TP}}{\mathrm{TP}+\mathrm{FP}},$$

(19) $$\mathrm{Rec}=\frac{\mathrm{TP}}{\mathrm{TP}+\mathrm{FN}},$$

(20) $$F1=2\times \frac{\mathrm{Pre}\times \mathrm{Rec}}{\mathrm{Pre}+\mathrm{Rec}},$$

where FN, FP, TN, and TP represent false negatives, false positives, true negatives, and true positives, respectively. In anomaly detection scenarios, we pay more attention to the accuracy of detecting real attacks or anomalies. Therefore, we focus more on F1 and recall.

(2) The training environment

We implement the proposed method on NVIDIA GeForce RTX 2060. For forecasting, the history input size is set to 40, and the length of the long forecasting is set to 10. For SWaT and WADI, the length of the embedding vector is set to 64 and 128. Meanwhile, the input embedding dimension of the general model is set to 256. The dimension of the fully connected network is set to 128. Both Encoder layers and Decoder layers are set to 2. Furthermore, to prevent overfitting, the dropout is set to 0.05.

### 4.3. Baselines

Our method has been compared with advanced multivariate time series anomaly detection methods, including

KNN [46]: K Nearest Neighbors employs the distance of each point to its kth nearest neighbor as a metric for scoring anomalies.

FB [47]: A Feature Bagging detector operates by training multiple detectors on different subsets of the dataset and then combining their detection scores through aggregation.

PCA [32]: Principal Component Analysis identifies a reduced-dimensional representation that preserves the majority of data variation. Anomaly detection is based on the deviation from this representation, quantified by the reconstruction error.

DAGMM [14]: Deep Autoencoding Gaussian Model merges deep Autoencoders with Gaussian mixture models to effectively capture data distribution complexities and offer a sophisticated approach to anomaly detection.

AE [48]: Autoencoders are composed of an encoding function and a decoding function that work together to reconstruct data points. The difference in fidelity between the original data and the reconstruction is utilized as the metric for anomaly detection.

LSTM- VAE [23]: LSTM-VAE combines LSTM network with VAE, enhancing anomaly detection in time series data by learning complex temporal patterns and distributions, thus effectively identifying unusual behaviors with high precision.

USAD [27]: USAD is an unsupervised anomaly detection technique that combines dual Autoencoders with adversarial training.

MAD-GAN [29]: A GAN model is trained on normal data, employing an LSTM-RNN-based discriminator in tandem with a reconstruction method to determine the anomaly rating for individual instances.

GDN [30]: GDN leverages graph-based embeddings and attention mechanisms to accurately detect anomalies in time series data by learning complex relationships and deviations within the data.

GTA [30]: GTA, standing for Graph Temporal Attention, is an advanced model that combines graph neural networks with temporal attention mechanisms to capture dynamic relationships and temporal dependencies in data for enhanced predictive analytics and anomaly detection.

TranAD [37]: TranAD, leveraging transformer architecture, enhances anomaly detection in time series data by capturing long-range dependencies and subtle patterns, thus offering improved precision in identifying irregularities.

STGAT-MAD [49]: STGAT-MAD integrates spatial and temporal graph attention mechanisms to effectively discern multivariate data anomalies, significantly enhancing the accuracy and efficiency of anomaly detection processes.

### 4.4. Accuracy

Table 3 and Table 4 show the performance of our approach and baseline methods on SWaT and WADI. Results in Table 3 are partially based on the work of [29]. To compare in a more comprehensive and integrated manner, in Table 4, we apply point-adjust way [8] to evaluate the proposed method. As shown in Table 3, optimal recall and F1 have been achieved with our method. In Table 4, the best Rec and F1 in WADI are demonstrated, achieving comparable performance in SWaT with our method.

In Table 3, we can see that these classical methods (PCA, KNN, and FB) do not perform as well as the deep learning methods (MAD-GAN, LSTM-VAE, AE, etc.). This shows that deep learning can better obtain the intrinsic information of time series and has a better anomaly detection effect. GDN has the best anomaly detection precision, which is 99.35% on SWaT, but its recall and F1 are not favorable. Thus, graph neural network can learn the time series features better, especially the normal data’s characteristics. It should be emphasized that optimality in recall and F1 have been achieved with our method based on short time series prediction and Informer. On SWaT, our method improves recall 1.26% and F1 1.23%, respectively, compared with the next best baseline. On WADI, our method improves recall 30.33% and F1 14.03%, respectively, compared with suboptimal baseline. However, precision is slightly inferior to other optimal values. However, in this scenario, we focus more on the recall and F1 metrics. Therefore, this result proves the superiority of our method.

**Table 4:** Anomaly detection performance of our method and baselines with point-adjust (precision: Pre (%); recall: Rec (%)).

Method	SWaT			WADI		
	**Pre (%)**	**Rec (%)**	**F1**	**Pre (%)**	**Rec (%)**	**F1**
USAD	**98.70**	74.02	0.85	64.51	32.20	0.47
GTA	94.83	88.10	**0.91**	**83.91**	83.61	0.84
TranAD	97.60	69.97	0.82	35.29	82.96	0.50
STGAT-MAD	84.10	**96.50**	0.90	79.70	91.00	0.85
Ours	94.97	86.70	0.91	82.06	**93.24**	**0.87**

In Table 4, we apply point-adjust approach to evaluate our method and baseline. On WADI, the best recall and F1 have been achieved with our method. On SWaT, we achieve excellent recall and F1. Additionally, comparable results have been achieved with our method in precision. As we introduced before, GTA and STGAT-MAD utilize graph attention, USAD applies Encoder and Decoder, and TranAD uses Transformer for time series outlier detection. Our scheme applies graph attention and Informer, where Informer contains Transformer and Encoder and Decoder. Therefore, these methods are used to display the characteristics of time series and achieve favorable results. It needs to be emphasized that excellent performance on the high-dimensional dataset is achieved with our method.

Compared to other baseline methods, our model demonstrates superior accuracy in anomaly detection. Through the learning of short- and long time series features, as well as mutual learning between them, our model achieves excellent performance in time series anomaly detection.

### 4.5. Ablation Experiment

To study each component of our method is indispensable; we excluded or replaced each component of the model and observed their performance. The experimental results are shown in Table 5.

To study the necessity of joint optimization, we remove $Loss_{z}$. To obtain the prediction, we calculate the average of the first value of long forecasting based on Informer and the output of short time series forecasting with GAT. When we exclude joint optimization, F1 decreases by 5.12% on SWaT and 38.30% on WADI, which indicates that using joint optimization, i.e., short time series prediction based on graph attention and long time series prediction based on Informer, is conducive to improving the accuracy of prediction.

To investigate the important role of Informer, we remove the long time series forecasting and utilize GAT and GRU for anomaly detection. The detection efficiency will decrease upon removal of Informer. When we compare the experimental results with GDN, we find that there is some improvement in the model of GAT after adding GRU, especially on the WADI dataset. This indicates the necessity of GRU to enhance the anomaly detection results. The experimental results show that utilizing Informer can effectively improve the anomaly detection results based on GAT. Long time series forecasting can assist and improve the anomaly detection effect.

To study the necessity of Informer, we employ traditional Transformer for long time series forecasting. When replacing Informer with traditional Transformer, the indicators of model anomaly detection show a decrease, indicating that, compared with the traditional Transformer, the model created by the Informer has a better anomaly detection performance.

To investigate the advantage of the proposed scheme in anomaly detection using long time series forecasting, LSTM is adopted instead of the Informer for reconstruction so that the method applies both prediction and reconstruction. When anomaly detection scheme based on GAT (prediction) and LSTM (reconstruction) is attempted, the effect is inferior to our method, which indicates that the effect of the method based on prediction and reconstruction may not be inferior to anomaly detection under the guidance of long-term prediction.

The experimental results show that replacing any component of the model leads to a decrease in performance, which proves the necessity of each component of the model and indicates that our model can more effectively carry out time series anomaly detection.

### 4.6. Effects of Model Parameters

In order to investigate the effect of model parameters, we select sliding window sizes and batch size to observe their influence.

**Figure 3:**
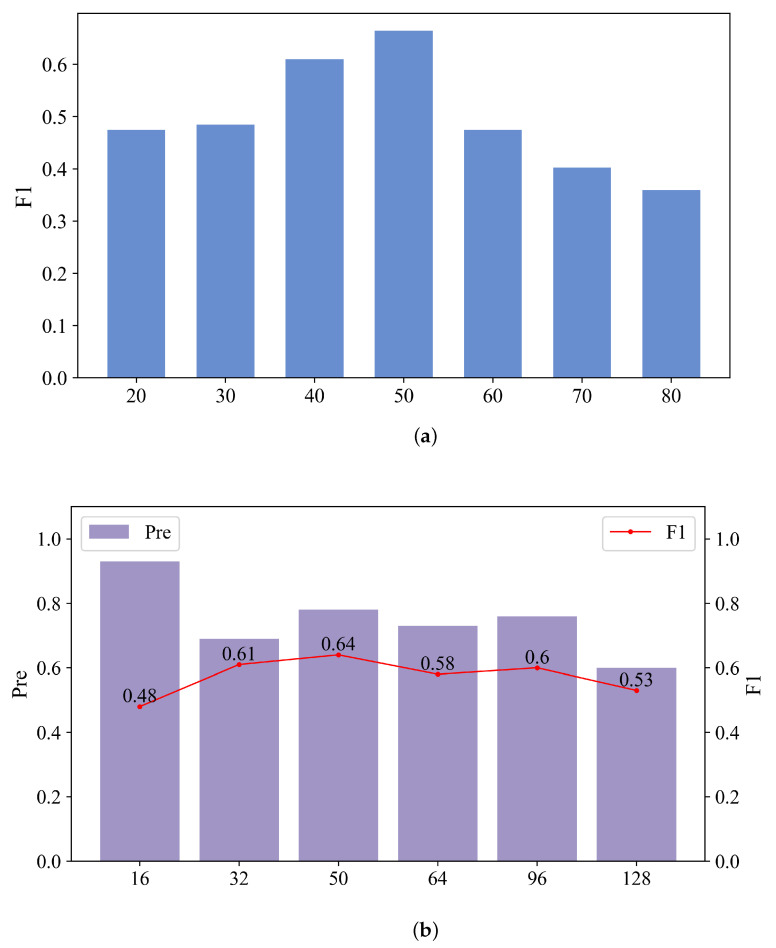
The performance of different sliding window size and batch size. (**a**) Sliding window size. (**b**) Batch size.

Figure 3a shows the F1 when setting different sliding window sizes while other parameters are fixed. As the window size increases, the model acquires more information, but the model’s performance does not improve. Setting the sliding window size to 40 or 50 may lead to better results in detecting anomalies in time series. We find that the sliding window should not be too small; otherwise, enough prior knowledge may not be obtained and should not be too large as well; otherwise, the key information may not be learned.

In Figure 3b, we try different batch sizes to observe the effects of Pre and F1. When the batch size is between 32 and 96, the accuracy is basically around 0.9, and the F1 is around 0.55. Therefore, batch size does not have a great impact on Pre and F1.

In general, setting different parameters to study the transformation of anomaly detection rate provides a reference for setting reasonable model parameters.

### 4.7. Situation of Abnormal Location

**Figure 4:**
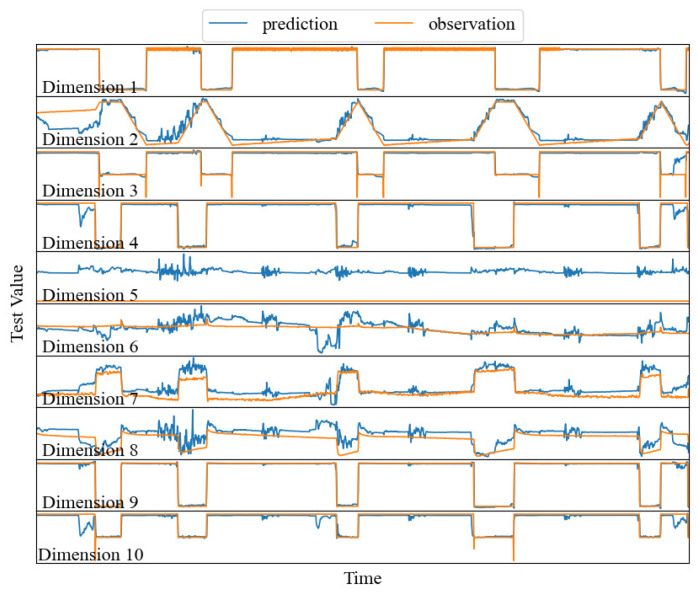
The predicted and true values of some sensors.

In order to observe the accuracy of our prediction, we present numerical curves of observed (true) and predict values for the first 10 dimensions of SWaT. In Figure 4, red represents the observed time series and blue represents the predicted time series. As we can see from Figure 4, the red and blue are essentially synchronized. This again demonstrates the effectiveness of our approach. We can judge which sensors are attacked based on the real values and predicted values.

**Figure 5:**
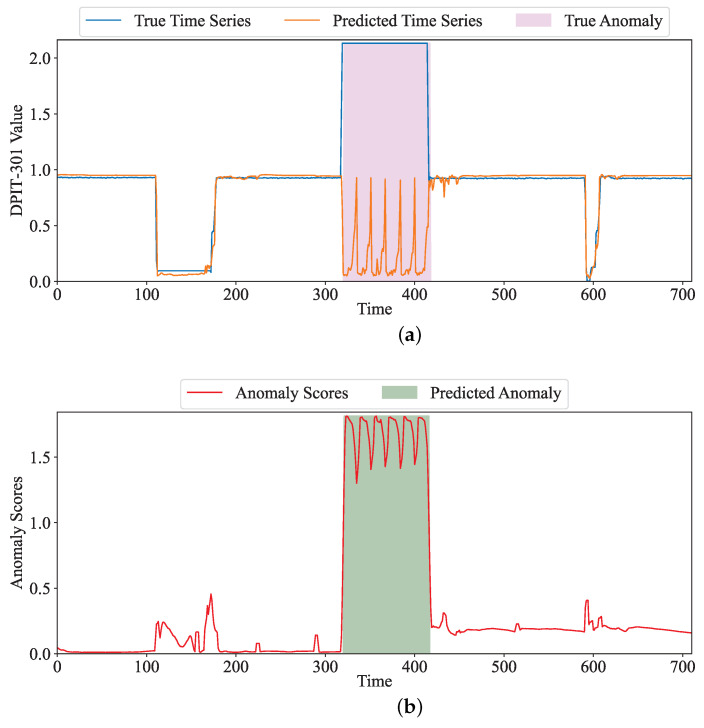
The true value, predicted value, and anomaly score of DPIT-301 in SWaT. (**a**) The true value and predicted value. (**b**) Anomaly score.

**Figure 6:**
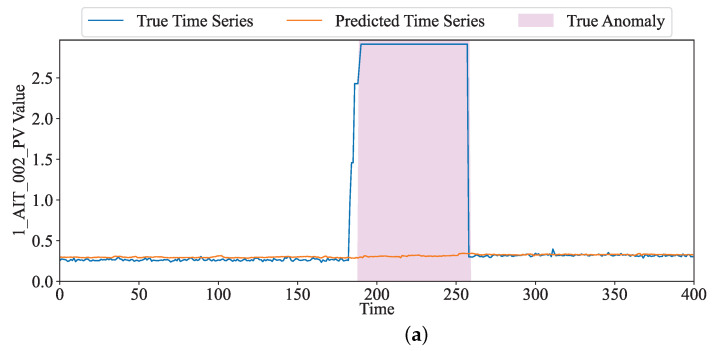

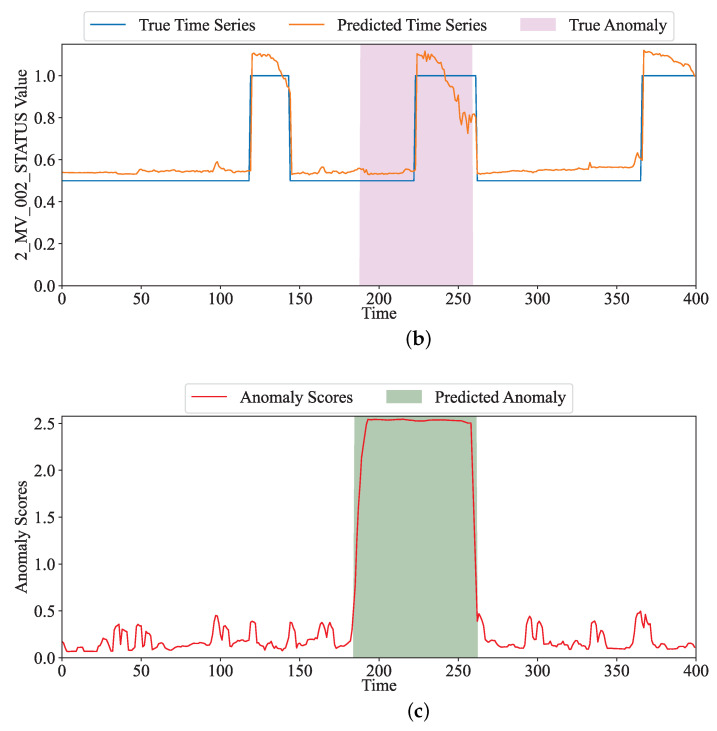
The true value, predicted value, and anomaly score of 1_AIT_002_PV and 2_MV_002_STATUS in WADI. (**a**) The The true value and predicted value of 1_AIT_002_PV. (**b**) The The true value and predicted value of 2_MV_002_STATUS. (**c**) Anomaly score.

As shown in Figure 5, we selected the dimension DPIP-301 in the SWaT dataset to show how to locate and interpret anomalies. In Figure 5a, the red curve represents observed values and the blue curve represents predicted data using our method. The pink block indicates the true anomalous time. Between 320 and 420 in the time dimension, there is an obvious difference between the real and predicted values, and we can see that the sensor may be under attack during this time. Figure 5b displays the anomaly score and the location of the predicted anomalies. Specifically, as shown in Figure 5b, the anomaly score increases significantly during this period. We find that the true anomalies and our judged anomalies are the same. In the real scenario, DPIT-301 was attacked during this period. The attacker modified the value of the sensor, which would lead to the wrong execution of its subsequent sensors, then causing security incidents. Figure 6 shows the observed and predicted values of our model for sensors 1_AIT_002_PV and 2_MV_002_STATUS, as well as the abnormal score and abnormal judgments given by the model during this period. From the attack description, we obtain that the attack lasted for 11.38 min; the attacker set 1_AIT_002_PV to 6 in order to supply contaminated water to the Elevated Reservoir tank. Meanwhile, 2_MV_003_STATUS was opened. In Figure 6b, our method can reflect the state of 2_MV_002_STATUS well, which is consistent with the actual state. However, although the attacker set the sensor 1_AIT_002_PV as 6 (as shown in Figure 6a); different values based on the inputs are predicted by means of the model. Due to the significant difference between observation and prediction, we obtained a larger abnormal score during this period, and the system was judged under attack during this time (as shown in Figure 6c).

Therefore, the expected behavior of each sensor is predicted with our model, and we obtain an anomaly score by forecasting and comparing it to the true values. The anomaly score is helpful to locate the anomaly. We can understand how the abnormal deviates from the expected and detect the anomaly in time. Meanwhile, we can explain the reasons for the anomaly determination and realize the determination of the anomaly.

## 5. Conclusions

Considering that the existing time series anomaly detection methods have not fully considered the interactions between time series of different dimensions, perform unsatisfactorily on high-dimensional data, and that prediction-based anomaly detection methods only focus on short-term time series forecasting while neglecting the influence of future trends, we propose a method based on GAT and Informer for time series anomaly detection. We apply GAT and GRU for short time series forecasting, Informer for long time series forecasting, and we apply long time series forecasting to aid short time series forecasting. The experiments show that, via our method, 0.704 recall and 0.82 F1 without point-adjust on SWaT have been obtained. On SWaT, our method improves recall 1.26% and F1 1.23%, respectively, compared with the best baseline. On WADI, our method improves recall 30.33% and F1 14.03%, respectively, compared with the baseline. It needs to be emphasized that our method can obtain the best recall and F1 on the high-dimensional dataset WADI compared with other advanced methods. In addition, our method not only has favorable accuracy but can also explain and locate anomalies. Our model improves the performance of time series anomaly detection by integrating the learning methods of two different models. However, this approach may lead to an increase in both model size and the number of parameters, which could impact its operational efficiency. To address this issue, we plan to develop a more lightweight version of the model using knowledge distillation technology without compromising model accuracy. This will facilitate efficient deployment across various environments in the future. In the future, we hope that the method can play a role in real ICS anomaly detection. At the same time, we are considering model optimization to obtain more abundant time series features and improve the accuracy of anomaly detection. 

## Figures and Tables

**Table 2:** Statistics of SWaT and WADI.

Dataset	#Train	#Test	#Dimensions	Anomalies
SWaT	47,515	44,986	51	11.97%
WADI	118,795	17,275	127	5.99%

**Table 3:** Anomaly detection performance of our method and baselines without point-adjust (precision: Pre (%); recall: Rec (%)).

Method	SWaT			WADI		
	**Pre (%)**	**Rec (%)**	**F1**	**Pre (%)**	**Rec (%)**	**F1**
KNN	7.83	7.83	0.08	7.76	7.75	0.08
FB	10.17	10.17	0.10	8.60	8.60	0.09
PCA	24.92	21.63	0.23	39.53	5.63	0.10
DAGMM	27.46	69.52	0.39	54.44	26.99	0.36
AE	72.63	52.63	0.61	34.35	34.35	0.34
LSTM-VAE	96.24	59.91	0.74	87.79	14.45	0.25
USAD	98.51	66.18	0.79	**99.47**	13.18	0.23
GDN	**99.35**	68.12	0.81	97.50	40.19	0.57
MAD-GAN	98.97	63.74	0.77	41.44	33.92	0.37
Ours	98.19	**70.40**	**0.82**	87.39	**52.38**	**0.65**

**Table 5:** Anomaly detection performance of our method and its variants without point-adjust.

Method	SWaT			WADI		
	**Pre (%)**	**Rec (%)**	**F1**	**Pre (%)**	**Rec (%)**	**F1**
ours	**98.19**	**70.40**	**0.82**	**87.39**	**52.38**	**0.65**
w/o Join	99.51	63.93	0.78	85.27	32.80	0.47
w/o Informer	98.19	67.16	0.79	80.85	51.79	0.63
w/Transformer	96.22	65.81	0.78	71.84	49.20	0.58
w/LSTM	98.06	67.11	0.79	83.54	39.36	0.53

## Data Availability

This research employed publicly available datasets for its experimental studies.

## Abbreviations

The following abbreviations are used in this manuscript:

ICS	Industrial Control System
GAT	Graph Attention Network
RNN	Recurrent Neural Network
CNN	Convolutional Neural Network
LSTM	Long Short-Term Memory
AE	Autoencoder
GDN	Graph Deviation Network
GRU	Gated Recurrent Unit
